# Household Exposure to Secondhand Smoke among Chinese Children: Status, Determinants, and Co-Exposures

**DOI:** 10.3390/ijerph17155524

**Published:** 2020-07-30

**Authors:** Muxing Xie, Chunrong Jia, Yawei Zhang, Beibei Wang, Ning Qin, Suzhen Cao, Liyun Zhao, Dongmei Yu, Xiaoli Duan

**Affiliations:** 1 School of Energy and Environmental Engineering, University of Science and Technology of Beijing, Beijing 100083, China; g20188207@xs.ustb.edu.cn (M.X.); wangbeibei723@ustb.edu.cn (B.W.); qinning@ustb.edu.cn (N.Q.); caosuzhen@ustb.edu.cn (S.C.); 2 School of Public Health, University of Memphis, Memphis, TN 38152, USA; 3 Department of Environmental Health Sciences, Yale School of Public Health, Yale University, New Haven, CT 06520, USA; yawei.zhang@yale.edu; 4 National Institute for Nutrition and Health, Chinese Center for Disease Control and Prevention, Beijing 100050, China; zhaoly@ninh.chinacdc.cn (L.Z.); yudm@ninh.chinacdc.cn (D.Y.)

**Keywords:** secondhand smoke, solid fuel smoke, school-age children, co-exposure, socioeconomic status

## Abstract

Smoking prevalence stays high among adults in China, which also makes children exposed to secondhand smoke (SHS) in their households. This study aimed to investigate the status of SHS exposure among Chinese children, identify the influencing factors, and determine “co-exposure” to tobacco and other smokes in households. A total of 41,439 children aged 6–17 years were recruited from 30 provinces in Mainland China through the first Chinese Environmental Exposure-Related Human Activity Model Survey for Children (CEERHAPS-C). Information regarding children’s demographics, socioeconomic status, and exposures to SHS and solid fuel smoke (SFS) in households was collected using a comprehensive questionnaire. Factors that affected exposures to household smokes were identified using multivariable logistic regressions. The overall prevalence of household SHS exposure was 41.7%, and the average duration was 14.7 ± 14.6 min/day among the exposed participants. Prevalence of household SHS exposure increased among children in older age groups and with parents in lower education levels. Among SHS-exposed children, 34% had co-exposure to SFS, and they had a significantly higher risk of co-exposure than non-SHS exposed children (odds ratio = 1.12, 95% confidence interval: 1.061, 1.162). The prevalence of household SHS exposure remains high among school-age children, suggesting the need to develop and implement smoking-free home programs.

## 1. Introduction

Smoking prevalence stays high in China, which inevitably leads to wide exposure to secondhand smoke (SHS) among children [1,2]. China is the largest producer and consumer of tobacco products in the world. Active smokers in China account for near 30% of the world’s total number of smokers, ranking the first in number [3]. The *2018 China Adult Tobacco Survey* showed that the smoking rate was 26.6% among people ages 15 and older, and the number of daily smokers was 269 million in China [2,4]. Exposure to SHS has become a serious public health issue in China, with 44.9% of Chinese adults having SHS exposure at home [1,2]. Children spend as much time at home as adults, and thus inevitably have SHS exposure if adults smoke at home. In China, the prevalence of SHS exposure is 80% among adolescents aged 12–15 [5]. The *2014 National Youth Tobacco Survey* showed that 72.9% of junior high school students saw someone smoking in homes, indoor and outdoor public places, or public transport [6]. The proportion of smokers in various types of venues, in the descending order, is 58.3% in outdoor public places, 57.2% in indoor public places, 44.4% in homes, and 37.9% in public transportation [6].

Many factors influence SHS exposure in children, such as lifestyle, smoking behaviors of teachers and parents, and awareness of the hazards [7,8,9,10]. Previous research has reported higher rates of active and passive smoking among adolescents globally [11]. Parental smoking is closely related to active and passive smoking among adolescents [5,10]. A survey of adolescent smoking in 68 countries has shown that parental smoking increases children’s risk of SHS exposure [5]. Socioeconomically disadvantaged children often bear higher SHS exposures [12,13]. Few Chinese studies have systematically examined the influencing factors [1,2,3]. Previous studies were focused on how children’s SHS exposure was affected by their own awareness and smoking behaviors of parents and teachers but lacked comprehensive identification of at-risk subpopulations [7,8,9,10]. 

Previous SHS research often ignored other smokes in households. One-third of the world’s population uses solid fuels derived from plant materials (biomass) or coal for cooking, heating, or lighting. Women and children living in severe poverty are most vulnerable to household air pollution [14]. About 50% of people in developing countries use coal and biomass as household energy in the form of wood, fertilizer, and crop residues, mainly in developing countries, including China, India, and many African countries [15]. In countries, such as China and Mongolia, solid fuels are still used for heating and cooking in a substantial portion of families, in particular, in rural areas [16,17]. Thus, children in those households are likely to have “co-exposure” to both tobacco and solid fuel smokes. The co-exposure to pollutant mixtures may pose higher health risks to children and has become an environmental health research priority [18]. No study has described the status and determinants of co-exposures to SHS and solid fuel smoke (SFS) in China. 

The objective of this study was to investigate the prevalence and determinants of SHS exposure among Chinese children. This study is part of a national survey of child exposure behavior patterns in China. We described the prevalence of SHS exposure and co-exposure to SHS and SFS among school-age children. We then identified the socio-demographic factors that influenced SHS exposures and co-exposure. We concluded with recommendations to reduce smoke exposures in children’s homes.

## 2. Materials and Methods 

### 2.1. Study Design and Population

This study utilized the data collected in the first Chinese Environmental Exposure-Related Human Activity Model Survey-Children (CEERHAPS-C). CEERHAPS-C was a cross-sectional national survey conducted from 2013 to 2016 to investigate factors affecting children’s exposure in China. The survey used a multi-stage stratified cluster random sampling method to recruit 41,439 children aged 6−17 from 316 schools in 30 provinces in mainland China (excluding Tibet). Specific sampling methods have been described in detail in previously published articles and books [16,19].

### 2.2. Data Collection

The CEERHAPS-C used a combination of questionnaires and field measurements to collect participant information. A panel of public health experts designed the questionnaire, which has been described previously [16,19]. The questionnaire consisted of three sections: (1) Information about the child’s family and parents, including location and urbanicity of the residence, annual family expenditure in Chinese Yuan, and household heating and cooking fuel types. (2) Information about the child’s parents, including age, ethnicity, education level, and occupation of the parents. It should be pointed out that there were no questions about the smoking status of the parents in the questionnaire, as this survey was primarily designed for children. (3) Questions about children’s behaviors and physical development, including children’s height/length (cm), weight (kg), water intake, nutrition intake, time-activity patterns, and exposure to indoor cooking and smoking smoke. In a face-to-face interview, the parent answered Section 1 and Section 2. For Section 3, the parent answered the questions if the child was younger than 9 years old, and the child aged 9 years and above (23.2% of all the participants) answered the questions by himself/herself. The participant who saw anyone smoke at or around his/her home in the past 7 days was defined as being exposed to SHS [19]. The interviewee also estimated the cumulative duration (min/day) of SHS exposure.

A quality assurance (QA) system was implemented throughout the survey. In the design stage, the questionnaire and protocols were designed by referring to existing validated survey plans and questionnaires and finalized after expert panel reviews, demonstrations, and a pilot study. The pilot study in Biyang validated the accuracy and feasibility of the questionnaire [20]. The National Institute for Nutrition and Food Safety, Chinese Center for Disease Control and Prevention reviewed and approved the survey protocols before the field investigation was launched (Ethics Review Number: 2013-018). In the preparation stage, national, provincial, and local training workshops were held for questionnaire administration and data entry. All the organizers, quality controllers, supervisors, and investigators must have participated in the training and passed the assessment before they could conduct the field data collection. The pre-survey training ensured uniformity among investigators in understanding and administration of the questionnaire. During the survey, questionnaires were completed in face-to-face interviews to ensure the response rate. Parallel questionnaires and measurements were performed among 5% of the participants to ensure repeatability. In the post-survey stage, the completed questionnaires underwent three levels of reviews: all the questionnaires were reviewed by onsite quality controllers and then were randomly selected by provincial and national supervisors. The survey response rate was 97.2%, the recovery rate was 100%, and the effective rate was 99.96%.

### 2.3. Variable Categorization and Data Analysis

In this study, we utilized data regarding (1) SHS and SFS exposures and (2) Socio-demographic information, including gender, age, ethnicity, urban and rural, nutritional intake, exercise time per week, region, family economic level, and mother’s education level. The SHS exposure status was dichotomous: exposed and non-exposed. The exposed group was further divided into three subgroups based on the SHS exposure duration: SHS1 (duration = 1–10 min/day), SHS2 (11–55 min/day), and SHS3 (≥56 min/day), representing mild, moderate, and severe exposure, respectively [21,22]. Indoor fuels were classified as solid fuel and clean fuel, and SFS exposure status was dichotomized as yes if the household was using solid fuel and no if using clean fuel. We then defined co-exposure as simultaneous exposure to both SHS and SFS.

Socio-demographic factors were categorized to facilitate the analyses. The per capita annual income was used to indicate the family’s economic level. The income was divided into seven levels: ≤5000 Yuan RMB, 5000–9999 Yuan, 10,000–14,999 Yuan, 15,000–19,999 Yuan, 20,000–24,999 Yuan, 25,000–29,999 Yuan, and ≥30,000 Yuan. Mother’s educational levels were classified into six levels: below primary school, primary school, middle school, high school, associate college degree, and bachelor’s degree and above. The geographic regions where participants lived were classified as South, North, Northeast, Southeast, Northwest, and Southwest China. 

We first computed the prevalence of SHS exposures and co-exposures by socio-demographic factors. We screened the potential influencing factors of SHS exposure and co-exposure using univariate logistic regression models (crude models). The crude models examined age, gender, urban and rural, ethnicity, region, family economic level, and mother’s educational level. The significant factors (*p* < 0.05) in crude models were then included in the multivariable logistic regression model. All hypothesis tests used *p* < 0.05 to indicate statistical significance. All the descriptive and regression analyses used survey weights to account for the multi-stage survey design. Regression models were run using the “survey” package in R (Ver 3.4.3, R Foundation for Statistical Computing, Vienna, Austria).

## 3. Results

### 3.1. Prevalence of Exposure and Co-Exposure

The overall prevalence of SHS exposure was 41.7% among all the survey participants, and the exposure duration averaged 14.7 ± 14.6 min/day among the exposed group (Table 1). Boys had a higher prevalence (43.1%) and slightly longer duration ( average 14.8 min/day among the exposed) than girls (40.1% and 14.7 min/day). The prevalence and duration of SHS exposure both increased in older age groups, e.g., the highest prevalence of 47.6% and the longest average duration of 17.1 min/day occurred in the 15–17 years group. SHS exposure displayed lower prevalence and shorter duration among children living in urban areas, with higher family income, and having a mother with a higher educational level. In terms of the geographic region, the SHS exposure prevalence was the highest in the Southwest (47.3%) and the lowest in the Northeast (33.0%). Children in households using solid fuels had higher prevalence and longer duration of SHS exposure, indicating higher chances of co-exposures.

A sizable fraction of children had co-exposure to both SHS and SFS in their households (Table 2). For those children with SHS exposure, 33.8% had SFS exposure, resulting in 14.1% of all the participants with co-exposure to SHS and SFS. This 14.1% could be further split into 8.5%, 4.8%, and 0.8% who had co-exposure to SFS and mild, moderate, or severe SHS exposures, respectively. As households used solid fuels for cooking and/or heating purposes, we further calculated the co-exposure prevalence by solid fuel uses. The prevalence of co-exposure to SHS and cooking SFS was 11.9% of all the participants, and that to SHS and heating SFS was 8.6%. Thus, 6.4% of all the participants had exposure to SHS and SFS from both cooking and heating.

### 3.2. Demographic and Social Determinants of SHS Exposure

Children’s SHS exposures significantly differed by demographic and social characteristics (Table 3). Girls had 10% more chances (95% CI: 1.05, 1.15) of having SHS exposure than boys, adjusted for other covariates. With increasing age, the chances of SHS exposure gradually increased (ORs = 1.10, 1.11, and 1.35, all significant). There was no significant difference in the risk of SHS exposure among different ethnic groups after adjusting for other variables (OR = 0.956, 95% CI: 0.892, 1.017). Comparing the six regions, the Northeast had the lowest exposure risk (OR = 0.746), and the Southwest had the highest (OR = 1.356) exposure risk, using North China as the reference in the full model. The risk of children’s SHS exposure stood out in households with incomes of 5000–15,000 Yuan. As the mother’s educational level increased, the risk of SHS exposure in children decreased (*p*-values < 0.05). The risk of SHS exposure for children whose mothers had an advanced degree decreased by 22% (95% CI: 0.695, 0.882) compared with children whose mothers had under-primary education.

### 3.3. Risks of Co-Exposure to SFS

Disproportionate exposure to household SFS existed in households with SHS (Table 4). Compared with children not exposed to SHS, the risk of SFS exposure increased by 12% (95% CI: 1.061, 1.162) among children exposed to SHS. After adjusting for gender, age, and urban/rural covariates, the risk of SFS exposure increased by 6.7% (95% CI: 1.012, 1.124). Further, the risk of cooking SFS exposure increased by 1.2% (1.002, 1.021), and the risk of heating SFS exposure increased by 12.6% (1.058, 1.198) among children with SHS exposure compared with those without SHS. This analysis suggests that children with SHS exposure had a higher risk of being simultaneously exposed to SFS in their households.

## 4. Discussion

### 4.1. Comparison with Previous Studies

The study, based on the first national children’s exposure factors survey, found that a high percentage (41.7%) of Chinese children had SHS exposure in their homes. Many studies reported the smoking status among young adolescents, and thus we used this age group for the comparison purpose. In our survey, the prevalence was 41.5% among children aged 12–15 years. The 2014 Chinese National Youth Tobacco Survey showed 44.4% of junior high school students (12–15 years) had SHS exposures at home [6]. The prevalence varied by provinces or cities: 42.1% in Beijing City [8], 43.0% in Henan Province [23], 39.8% in Guangxi Province [24], 45.3% in Chengdu City [7], and 34.5% in Guangzhou City [9]. Our results agreed with the statistics from these national and regional surveys, indicating the good national representativeness of this survey.

Internationally, the prevalence of SHS exposure is generally high among adolescents aged 12–15. The 1999–2005 Global Youth Tobacco Survey showed that the prevalence was 44.1% at home and 54.2% in public places for adolescents 13–15 years old [11]. In a study of adolescent smoking in 68 low-income and middle-income countries, the overall average prevalence of SHS exposure was 55.9% among adolescents aged 12–15 years, ranging from 16.4% in Tajikistan to 85.4% in Indonesia [5]. The SHS exposure prevalence among Chinese children was in the middle of this range. Differences in the prevalence could be attributed to differences in demographics, economic levels, lifestyles, and dietary patterns between countries [2,5,6,11].

### 4.2. Determinants of Children’s SHS Exposure

The determinants of children’s SHS exposure identified in this study confirmed those reported in the literature. A 68-country survey showed that female and older children had higher risks of SHS exposure [5], as they were more likely to contact smokers [5,7,25]. Children are also more likely to contact SHS if their peers, siblings, teachers, parents, or guardians smoke [7,8,9,23,24]. The wide smoking bans in closed public spaces may push adult smokers to smoke at home [26]. A 31-country study showed that 88% of smoking parents continued to smoke at home, and 80% of parents smoked at home when their children were present [27]. 

Multiple reasons could explain the higher prevalence of SHS exposure in older children. First, parents tend to restrict their smoking behaviors to protect young children; however, this tendency diminishes for older children [7,8]. Second, older children spend more time in indoor environments because of academic stress [28] and more screen time [29]. Combining with their higher mobility, they have more opportunities to be exposed to SHS in different places at home. Third, the active smoking prevalence increases in older children, increasing the chance of SHS exposure in peers [5]. 

The influence of mothers’ education levels on SHS exposure could be explained by their own active smoking status, awareness of SHS hazards, and their close relationship with children. Women with lower education levels are more likely to smoke, as reported by studies in Brazil [30], Swede [31], Spain [13], Mid-East [21], and China [4]. Well-educated parents are aware of the hazards of tobacco smoking and SHS, as well as the need for prevention [7,8,9]. Mothers’ smoking behaviors and awareness have greater impacts as they spend more time with children than fathers [32].

Household income and SHS exposure did not display a simple discordant relationship in this study. The prevalence of SHS exposure peaked in low-income families (5000–15,000 Yuan) but decreased in the poorest (<5000 Yuan) and higher-income (≥15,000 Yuan) families. The low-income parents restrict their smoking due to their affordability [2,5,24], e.g., smokers in Shanghai, China, tend to lower smoking duration and intensity, given an increase in tobacco retail price [33]. Previous studies have reported a high prevalence of active and passive smoking in low-income populations [34]. A U.S. study reported people in lower socioeconomic status had increased nicotine dependence, cigarettes per day, and nicotine exposure [35]. In China, blue-collar smokers have a significantly longer smoking duration than white-collar smokers [36]. Our findings provide evidence that supports education and tobacco price policy as effective tobacco control measures.

### 4.3. Study Limitations

The strengths of this study included a large nationally representative child sample, effective quality control for onsite measurements, high response rates, information on multiple exposures, and comprehensive socio-demographic factors. Of course, there were limitations and uncertainties in the survey. Smoke exposure was self-reported without using objective tobacco smoke indicators, such as environmental nicotine and blood cotinine. However, tobacco research has confirmed the reliability of self-reported smoking status, and questionnaires can collect long-term SHS exposure information [20,37]. Tobacco smoke biomarkers also have limitations, e.g., urine cotinine measurements can only reflect short-term tobacco smoke exposure, and blood cotinine is not a good indicator of SHS exposure [16,38]. Nicotine and cotinine accumulate and remain stable in hair [39], making them appropriate biomarkers of long-term exposure to SHS [40,41]. Future studies may adopt hair cotinine as an objective SHS biomarker. Parents might have misreported their smoking status due to social pressures on smoking, leading to exposure misclassification [42]. The questionnaire did not cover the smoking status of parents and other family members, the occupation of the parents, or the level of children and parents’ understanding of tobacco harm, which have been reported to impact SHS exposure [5,7,8,9].

### 4.4. Implications for the “Smoking-Free Home Program”

China adopted WHO’s *Framework Convention on Tobacco Control* [43,44] in 2003. Under this framework, China committed to banning tobacco smoking in indoor workplaces, public transportation, and public indoor spaces within three years. In 2011, China’s Ministry of Health enacted the “Implementation Rules for Sanitation Management Regulations in Public Places” that completely banned tobacco smoking in ten types of public places, such as theaters, internet cafes, and gymnasiums [45]. There has not been a smoking-free policy for homes, although several interventions utilized the “no smoking at home” strategy [46].

This study calls for promoting the “smoking-free homes program (SFHP)”, given the high prevalence of SHS exposure [47,48] and a long time spent at home among children [49]. Smoke-free legislation has proved to bring health substantial benefits to children [50,51]. Children’s SHS exposure in public places is expected to be minimal due to smoking restrictions in most public places. Thus, promoting the SFHP is the most effective way to reduce or eliminate SHS exposure among children. Both literature and our findings suggest the successful adoption of SFHP should be focused on smoking behaviors and awareness among parents and older adolescents. SHS exposure can be reduced by formulating smoking rules for parents and caregivers [5,7,8,9]. For example, eight states in the US have laws prohibiting smoking in front of children and adolescents at home or in vehicle cabins [52]. Smoking parents are more likely to have children who smoke regularly, and thus interventions should target adults who smoke by educating them about the adverse health effects on children [5,53]. The concept of a “home environment” should also be extended to private vehicles [54]. Our results showed that 1/3 of SHS-exposed children were also exposed to SFS, and the SFS exposure risks were elevated in smoking homes. These co-exposure findings suggest the need to reduce SFS sources when implementing SFHPs, e.g., clean fuel substitution and stove modification [55,56].

## 5. Conclusions

This is the first national-level population-based survey on household exposure to secondhand smoke (SHS) and solid fuel smoke (SFS) among school-age children in China. The overall prevalence of SHS exposure was 41.7%, 33.8% of which were also exposed to SFS. Children with SHS exposure had a higher risk of co-exposure to SFS, compared with children without SHS exposure (OR = 1.067, 95% CI: 1.012, 1.124). Children’s SHS exposure was determined by gender, age, region, family income level, and mother’s education level. Reduction of household smoke exposure should consider adopting “smoke-free homes programs (SFHPs)” integrated with control of solid fuel uses.

## Figures and Tables

**Table 1:** Secondhand smoke (SHS) exposure status by subgroups of the study population.

Characteristics	*n*	Secondhand Smoke (SHS) Exposure Status		
		No	Ever (95% CI)	Duration (min/day) ^1^
**Total**	40,186	58.3%	41.7% (40.9–42.3%)	14.7 (14.6)
Gender ***				
Boys	19,415	59.9%	40.1% (39.0–41.0%)	14.8 (15.2)
Girls	20,771	56.9%	43.1% (42.2–43.9%)	14.7 (14.0)
Age group ***				
6~<9 years	9304	62.9%	37.1% (35.9–38.2%)	13.1 (11.9)
9~<12 years	10,962	59.4%	40.6% (39.5–41.8%)	13.0 (12.3)
12~<15 years	10,410	58.5%	41.5% (40.2–42.5%)	15.3 (15.8)
15~<18 years	9510	52.4%	47.6% (46.2–48.9%)	17.1 (16.7)
**Urbanicity**				
Urban	14,427	58.8%	41.2% (40.0–42.1%)	14.5 (15.4)
Rural	25,759	58.1%	41.9% (41.2–42.7%)	14.8 (14.1)
Ethnicity *				
Han ethnicity	34,472	58.5%	41.5% (39.9–41.5%)	14.6 (14.6)
Other ethnicities	5399	56.8%	43.2% (41.5–44.9%)	15.5 (14.5)
Region ***				
North	8035	60.2%	39.8% (38.8–40.9%)	14.9 (15.1)
East	10,595	60.4%	39.6% (38.2–40.9%)	14.1 (14.4)
South	6885	52.8%	47.2% (45.5–48.9%)	14.4 (14.2)
Northwest	5002	59.1%	40.9% (39.6–42.3%)	16.7 (15.3)
Northeast	3731	67.0%	33.0% (30.6–35.6%)	15.6 (15.4)
Southwest	5938	52.7%	47.3% (45.2–49.3%)	14.1 (13.3)
Annual income (Yuan/capita) ***				
<5000	2367	59.9%	40.1% (38.5–42.7%)	15.3 (15.2)
5000–9999	2701	55.7%	44.3% (41.6–46.8%)	15.8 (15.3)
10,000–14,999	3242	55.8%	44.2% (41.7–46.8%)	15.3 (14.7)
15,000–19,999	2278	59.3%	40.7% (37.8–43.6%)	15.0 (14.3)
20,000–24,999	1915	60.4%	39.6% (37.3–42.0%)	14.4 (15.1)
25,000–29,999	1461	59.2%	40.8% (38.8–43.9%)	13.5 (13.6)
≥30,000	4701	61.9%	38.1% (36.1–40.1%)	13.8 (13.9)
Educational level of the mother ***				
Below primary school	2101	56.2%	43.8% (41.1–46.5%)	17.4 (16.9)
Primary school	6715	54.5%	45.5% (44.1–46.9%)	15.8 (15.3)
Middle school	15,588	56.5%	43.5% (42.2–44.8%)	15.2 (14.6)
High school	8574	60.6%	39.4% (38.0–40.1%)	13.8 (13.9)
Associate college	3799	62.3%	37.7% (35.6–39.9%)	12.8 (13.2)
Bachelor and above	3136	66.0%	34.0% (31.8–36.2%)	12.3 (12.7)
**Household** fuel type ***				
Clean fuel	28,136	60.2%	39.8% (39.0–40.6%)	14.1 (14.1)
Solid fuel	12,050	57.1%	42.9% (41.3−44.3%)	16.2 (15.3)

Notes: 1. Secondhand smoke duration among the exposed group. * *p* < 0.05; ** *p* < 0.01; *** *p* < 0.001.

**Table 2:** Percentages of children with co-exposure to SHS and solid fuel smoke (SFS).

		*n*	NSHS	SHS	SHS1	SHS2	SHS3
**SFS**	No	28,136	40.4%	26.7%	17.9%	7.8%	1.0%
	Yes	12,050	18.7%	14.1%	8.5%	4.8%	0.8%
**Heating**	No	32,179	47.3%	33.1%	21.7%	10.0%	1.4%
**SFS**	Yes	7872	11.0%	8.7%	5.1%	3.1%	0.5%
**Cooking**	No	21,644	42.8%	28.9%	19.2%	8.6%	1.1%
**SFS**	Yes	8513	16.3%	11.9%	7.2%	4.0%	0.7%

Notes: NSHS: No secondhand smoke exposure. SHS is further classified as mild (SHS1), moderate (SHS2), and severe (SHS3) exposures, based on the SHS duration. SFS: Solid fuel smoke. The proportion of exposed persons is expressed as a percentage (%) of all the participants.

**Table 3:** Effects of socio-demographic factors on children’s exposure to SHS.

Factors	Crude Model	Full Model ^1^
	OR (95% CI) ^2^	OR (95% CI)
**Gender (Boys) ^3^**	*n* = 40,186	
Girls	1.112 (1.058, 1.149) ***	1.101 (1.046, 1.148) ***
**Age** **group (6** **−** **8 years)**	*n* = 40,186	
9–11 years	1.135 (1.070, 1.203) ***	1.101 (1.038, 1.173) **
12–14 years	1.167 (1.101, 1.232) ***	1.112 (1.045, 1.183) **
15–17 years	1.426 (1.383, 1.507) ***	1.345 (1.257, 1.436) ***
**R** **egion (North China)**	*n* = 40,186	
East China	0.982 (0.924, 1.039)	0.991 (0.934, 1.052)
South China	1.258 (1.247, 1.398) **	1.340 (1.255, 1.430) **
Northwest China	0.978 (0.945, 1.016)	1.027 (0.956, 1.104)
Northeast China	0.676 (0.583, 0.798) ***	0.746 (0.687, 0.809) ***
Southwest China	1.232 (1.169, 1.352) ***	1.356 (1.267, 1.451) ***
**Annual income (<5000 Yuan)**	*n* = 39,755	
5000–9999 Yuan	1.202 (1.071, 1.349) **	1.223 (1.093, 1.369) *
10,000–14,999 Yuan	1.221 (1.093, 1.364) ***	1.212 (1.088, 1.350) **
15,000−19,999 Yuan	1.115 (0.988, 1.259)	1.064 (0.946, 1.197)
20,000−24,999 Yuan	1.050 (0.925, 1.193)	1.015 (0.897, 1.149)
25,000−29,999 Yuan	1.141 (0.995, 1.309)	1.074 (0.940, 1.228)
≥30,000 Yuan	1.017 (0.915, 1.132)	0.974 (0.880, 1.079)
**Mother’s educational level** (Under primary school)	*n* = 39,913	
Primary school	1.077 (0.975, 1.191)	1.079 (0.977, 1.191)
Junior middle school	1.068 (0.971, 1.173)	1.044 (0.951, 1.145)
Senior high school	0.911 (0.825, 1.006)	0.898 (0.815, 0.991) *
Junior college	0.880 (0.787, 0.985) *	0.860 (0.771, 0.960) *
Universities or above	0.770 (0.685, 0.867) ***	0.739 (0.659, 0.830) **
**Urban or rural (urban)**	*n* = 40,186	Not included
Rural	1.022 (0.978, 1.063)	
**Ethnicity (Han ethnicity)**	*n* = 39,871	Not included
Other ethnicities	1.049 (0.902, 1.116)	

^1^ The full model was adjusted for age, gender, ethnicity, geographical regions, mother’s education level, income level. Only the variables showing significant effects in crude models were included in the full model. ^2^ OR: Coefficients of demographic and social factors covariates in the weighted logistic regression model with SHS exposure risk as the dependent variable. CI: confidence interval. ^3^ The group in the parentheses is the reference group. * *p* < 0.05; ** *p* < 0.01; *** *p* < 0.001.

**Table 4:** Risk of co-exposure to SFS among children with SHS exposure.

Exposure	Crude Model	Full Model ^1^
	OR (95% CI) ^2^	OR (95% CI)
**SFS exposure (NSHS) ^3^**	*n* = 40,186	*n* = 40,186
SHS	1.118 (1.061, 1.162) ***	1.067 (1.012, 1.124) *
**Cooking (NSHS)**	*n* = 30,157	*n* = 30,157
SHS	1.079 (1.017, 1.122) **	1.012 (1.002, 1.021) *
**Heating (NSHS)**	*n* = 40,051	*n* = 40,051
SHS	1.122 (1.070, 1.183) ***	1.126 (1.058, 1.198) ***

^1^ The full models were adjusted for age, gender, urban or rural, ethnicity, geographical regions, mother’s education level, income level. ^2^ OR: Coefficients of demographic and social factors covariates in the weighted logistic regression model with SHS exposure risk as the dependent variable. CI: confidence interval. ^3^ The group in the parentheses is the reference group. SFS: Solid fuel smoke; NSHS: No exposure to secondhand smoke; SHS: Exposure to secondhand smoke. * *p* < 0.05; ** *p* < 0.01; *** *p* < 0.001.
